# European Code Against Cancer, 5th edition – cancer‐causing infections and related interventions

**DOI:** 10.1002/1878-0261.70172

**Published:** 2026-01-16

**Authors:** Catharina Johanna Alberts, Paul Bloem, Silvia de Sanjosé, Sophie Grabar, Marcis Leja, Peter Malfertheiner, Mojca Matičič, Francis Mégraud, Francesco Negro, Martyn Plummer, Maarten Schim van der Loeff, Graciela Balbín, Josefina Salazar, Hajo Zeeb, Ariadna Feliu, Erica D'Souza, David Ritchie, Carolina Espina, Silvia Franceschi

**Affiliations:** ^1^ Department of Infectious Diseases Public Health Service (GGD) Amsterdam The Netherlands; ^2^ Department of Epidemiology and Data Science Amsterdam UMC The Netherlands; ^3^ World Health Organization Geneva Switzerland; ^4^ ISGlobal Barcelona Spain; ^5^ Sorbonne Université, INSERM, Institut Pierre Louis d'Epidémiologie et de Santé Publique, Assistance Publique‐Hopitaux de Paris, Hôpital St Antoine Paris France; ^6^ Institute of Clinical and Preventive Medicine University of Latvia Riga Latvia; ^7^ Medical Department II, University Clinic Ludwig Maximilian University München Germany; ^8^ Clinic for Infectious Diseases University Medical Centre Ljubljana Ljubljana Slovenia; ^9^ Faculty of Medicine University of Ljubljana Slovenia; ^10^ INSERM U1312 BRIC, University of Bordeaux France; ^11^ University Hospitals of Geneva Switzerland; ^12^ University of Warwick UK; ^13^ Amsterdam University Medical Centre The Netherlands; ^14^ Iberoamerican Cochrane Centre Biomedical Research Institute Sant Pau (IIB Sant Pau) Barcelona Spain; ^15^ Leibniz‐Institute for Prevention Research and Epidemiology‐BIPS Bremen Germany; ^16^ Environmental and Lifestyle Epidemiology Branch International Agency for Research on Cancer Lyon France; ^17^ Department of Primary Care and Public Health, School of Public Health Imperial College London London UK; ^18^ Centro di Riferimento Oncologico di Aviano (CRO) IRCCS Aviano Italy

**Keywords:** cancer, European Code Against Cancer, (HBV and HIV), *Helicobacter pylori*, hepatitis B virus, hepatitis C virus, human immunodeficiency virus, human papillomavirus

## Abstract

The main infections that cause cancer in the European Union (EU) are *Helicobacter pylori* (*H. pylori*), human papillomavirus (HPV), hepatitis B virus (HBV), hepatitis C virus (HCV) and human immunodeficiency virus (HIV). Altogether, in 2022, these infections accounted for ~ 5% of all cancers in the EU, mainly of the stomach, cervix uteri and liver. The largest burden of infection‐caused cancers was found in the south of the EU and near the eastern border. Substantial progress in the efficacy of interventions against these infections has been made since the release of the 4th edition of the European Code Against Cancer in 2015. Cancers due to infections can increasingly be prevented by prophylactic vaccines (HPV and HBV) and/or prompt diagnosis and treatment that can either cure (HCV and *H. pylori*) or slow down the infection (HBV and HIV), thus substantially reducing disease risk. Tools to tackle carcinogenic infections are also increasingly accessible and affordable in the EU, but their implementation is slow. Public awareness, political will and cost‐effective protocols are necessary to establish large programmes of vaccination or testing and treatment. Progress monitoring, as well as avoiding disinformation and stigma, is crucial to ensure that advances in medical progress are fully leveraged. The recently published 5th edition of the European Code Against Cancer therefore recommends: (1) vaccinate girls and boys against HBV and HPV at the age recommended in your country; (2) take part in testing and treatment for HBV and HCV, HIV and *H. pylori*, as recommended in your country.

## Graphical abstract

The 5th edition of the European Code Against Cancer (ECAC5) contains 14 recommendations on cancer prevention. Here, we update the cancer prevention recommendations related to cancer‐causing infections, namely *H. pylori*, HPV, HBV, HCV and HIV, positioned as recommendation number 12. ECAC5 recommends to: (1) vaccinate girls and boys against HBV and HPV at the age recommended in your country; and (2) take part in testing and treatment for HBV and HCV, HIV and *H. pylori*, as recommended in your country. Policy‐makers should expand vaccination and improve access to testing and treatment.

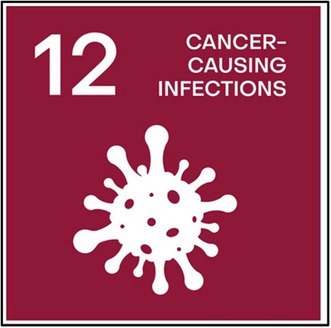

**Abbreviations**
AF: attributable fraction
aHR: adjusted hazard ratio
ART: antiretroviral treatment
ASR: age‐standardized Incidence Rate
DAAs: direct‐acting antivirals
EBV: Epstein–Barr Virus
ECAC: European Code Against Cancer, 4th edition
ECAC4: European Code Against Cancer, 5th edition
ECAC5: European Code Against Cancer
ECDC: European Centre for Disease Prevention and Control
EEA: European Economic Area
EU: European Union, 27 countries are part of the European Union: Austria, Belgium, Bulgaria, Croatia, Cyprus, Czechia, Denmark, Estonia, Finland, France, Germany, Greece, Hungary, Ireland, Italy, Latvia, Lithuania, Luxembourg, Malta, Netherlands, Poland, Portugal, Romania, Slovakia, Slovenia, Spain, Sweden
*H. pylori*
: *Helicobacter pylori*
HBV: hepatitis B virus (HBV)
HCC: hepatocellular carcinoma
HCV: hepatitis C virus (HCV)
HHV8: human gamma herpesvirus 8
HIV: human immunodeficiency virus (HIV)
HPV: human papillomavirus
IARC: International Agency for Research on Cancer
ICC: invasive cervical cancer
IFN: interferon‐based regimens
KS: Kaposi's sarcoma
LAC Code: Latin American and the Caribbean Code
MSM: men who have sex with men
NHL: non‐Hodgkin lymphoma
PLWH: people living with HIV
PPI: proton pump inhibitor
PWID: people who inject drugs
RCT: randomized clinical trials
RNA: ribonucleic acid
RR: relative risk
SVR: sustained virologic response
UK: United Kingdom

## 1 Introduction

**Fig. 1:**
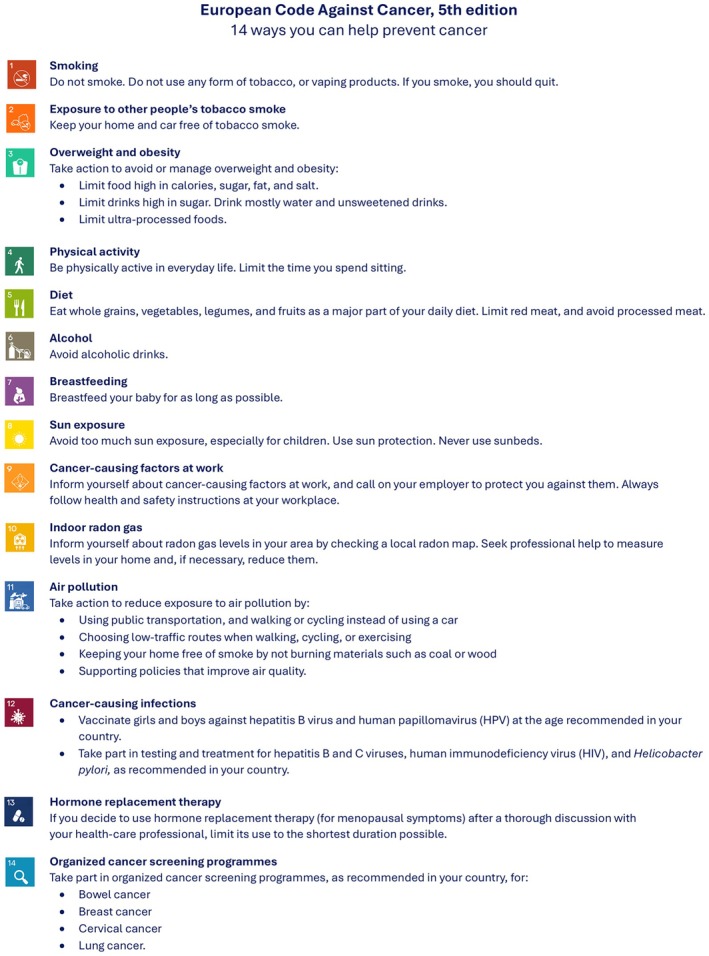
European Code Against Cancer, 5th edition: recommendations for individuals. The 14 recommendations of the European Code Against Cancer, 5th edition (ECAC5) adopted by the Scientific Committee of the ECAC5 project. © 2026 International Agency for Research on Cancer / WHO. Used with permission.

The 5th edition of the European Code Against Cancer (ECAC) [1] (Fig. 1) builds on the previous edition [2], under the umbrella of the World Code Against Cancer Framework [3]. For the first time, ECAC5 is aimed not only at individuals but also at policymakers (Annex S1). This paper reviews and integrates the latest scientific evidence on cancer‐causing infections and related interventions using the International Agency for Research on Cancer (IARC) methodology described elsewhere [4], and presents the updated ECAC5 recommendations for the general public and the new recommendations on population‐level measures that may reinforce the recommendations for individuals, along with a summary of the supporting evidence.

### 1.1 Most important cancer‐causing infections in the European Union (EU)

Eleven infectious agents have been classified by IARC as Group 1 carcinogenic to humans [5, 6, 7, 8, 9]. In the present report, we focussed on infectious agents that cause the majority of cancers in the EU and in which malignant evolution can be avoided by means of vaccination or prompt diagnosis and treatment, that is human papillomavirus (HPV), hepatitis B virus (HBV), hepatitis C virus (HCV), human immunodeficiency virus (HIV) and a bacterium, *Helicobacter pylori* (*H. pylori*). Altogether, they account for more than 90% of infection‐related cancers worldwide [10]. If these infections become chronic, they can cause cancer through different mechanisms: (a) direct damage to the cell DNA, (e.g. HBV and HPV); (b) progressive inflammation (e.g. HBV, HCV and *H. pylori*); and (c) immunosuppression that, in turn, increases the risk of cancer by other infections (e.g. HIV) [11]. Additional agents that are classified as carcinogenic to humans by IARC (Epstein–Barr virus; Kaposi sarcoma herpesvirus; Human T‐cell Lymphotropic virus type 1; and a few parasites [11]) are much less present in the EU than the five aforementioned infections and are therefore not discussed here.

In the large family of mucosal DNA HPV viruses, HPV16 and HPV18 cause about 80% of invasive cervical cancer (ICC) cases, and together with HPV31, 33, 45, 52 and 58, they account for 95% of all ICC in Europe [12]. While HPV‐related tumours are predominantly occurring in women, men can also develop HPV‐related cancers. Extensive epidemiological studies, molecular biology research and mechanistic studies have consistently demonstrated that HPVs cause varying proportions of cancer of the anus, vulva, vagina, penis and oropharynx, most commonly associated with HPV16, among other types [13, 14]. The main mode of transmission of mucosal HPVs is through sexual/intimate contacts.

HBV (a DNA virus) and HCV (an RNA virus) are major causes of chronic hepatitis that are usually asymptomatic but can progress to cirrhosis in 5–10% of those being infected for more than 20 years [15, 16]. Once cirrhosis is established, hepatocellular carcinoma (HCC) develops with a risk of 2–5% per year [16]. Progression of liver disease in individuals co‐infected with both HBV and HCV, or HBV and hepatitis D virus (HDV) is faster than with a single infection only [17]. Currently, contaminated blood presents the major source of acute HCV infection in the EU, primarily via injecting drugs, followed by nosocomial exposure, which also presents a considerable risk for acute HBV infection. Sexual transmission is an important route of HBV transmission (i.e. among heterosexuals and men who have sex with men) but it can also occur for HCV infection (i.e. among men who have sex with men) [5].

Due to HIV‐related immunodeficiency and immune dysfunction, HIV infection is a significant risk factor for cancers, particularly those caused by Epstein–Barr virus, Kaposi sarcoma herpesvirus and HPV [18]. In the EU, 0.4–2.2% of ICC are attributed to HIV [19]. The main modes of HIV transmission are sexual intercourse, exposure to contaminated blood and from mother to child. Increased cancer risk in people living with HIV (PLWH) is also related to a disproportional high prevalence of oncogenic viruses other than HIV and behavioural risk factors, for example tobacco, alcohol and multiple partners, among PLWH.

*Helicobacter pylori* is the primary cause of gastric cancer worldwide, confirmed by many epidemiological studies, clinical intervention trials, animal experiments, biological plausibility [20]. Chronic *H. pylori* infection induces persistent inflammation of the gastric mucosa that can progress to atrophic gastritis and intestinal metaplasia and ultimately gastric cancer [20]. The main modes of *H. pylori* transmission are oral–oral, fecal–oral and gastric‐oral (i.e. via vomiting events) routes, and most infections are acquired during childhood and persist lifelong if not treated. In Europe, the population attributable fraction of *H. pylori* infection is large in cancer of the noncardia (89%) but low in the cardia types [21, 22]. New consensus reports suggest a role of *H. pylori* at the gastro‐oesophageal junction [22].

### 1.2 Cancer burden attributable to cancer‐associated infections in the European Union

The number of cancer cases attributable to infections in the present paper refers to the 27 countries (Austria, Belgium, Bulgaria, Croatia, Cyprus, Czechia, Denmark, Estonia, Finland, France, Germany, Greece, Hungary, Ireland, Italy, Latvia, Lithuania, Luxembourg, Malta, Netherlands, Poland, Portugal, Romania, Slovakia, Slovenia, Spain and Sweden) that belong to the European Union (EU) whose total population is about 450 million. Reported age‐standardized Incidence Rates (ASRs) are different and generally lower than in the even broader United Nations' European Region. We estimated ASRs and population attributable fraction, that is the proportion of cancer cases that would have been prevented in a population if the infection(s) had been avoided or successfully treated. To do so, we used previously published methodology [10, 21], cancer estimates from GLOBOCAN 2022 [23] and age‐standardization using the EU population [24]. Briefly, the attributable fraction (AF) for each combination of agent and cancer site was calculated from the relative risk (RR) and the prevalence in cases (pc) using the formula AF = pc*(RR‐1)/RR. Attributable fractions were taken from de Martel et al. [21] and were applied to cancer incidence data from GLOBOCAN 2022 [23] to estimate the number of cases attributable to HPV, HBV, HCV and *H. pylori* infections by country, age and sex in 2022 [25]. Notably, a 89% attributable fraction was used for noncardia gastric cancer while a 0% was used for cancer in the cardia in the EU. ASR attributable to HIV is not estimated separately here, as the virus acts in combination with other carcinogenic viruses. This will avoid double counting the cancer burden attributable to both infections.

**Fig. 2:**
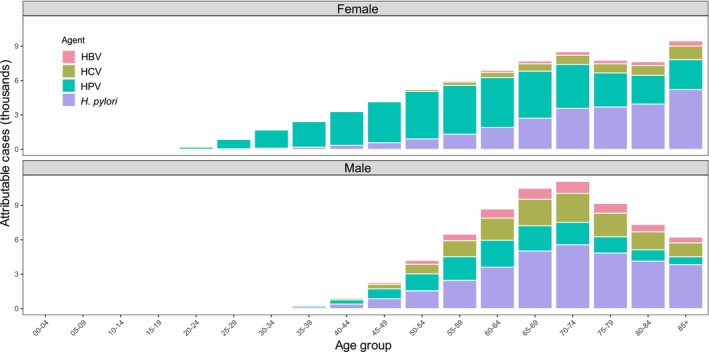
Number of cancer cases attributable to *Helicobacter pylori* (*H. pylori*), human papillomavirus (HPV), hepatitis C virus (HCV), and hepatitis B virus (HBV) in the European Union by sex and age group. The figure provides the numbers of cancer cases attributable to infections in 27 EU countries. Attributable fractions [21] were applied to cancer incidence data from GLOBOCAN 2022 [23] to estimate the number of cases attributable to HPV, HBV, HCV, and *H. pylori* infections by country, age, and sex in 2022 [25].

Overall, ~5% of all cancer cases in the EU in 2022 were attributable to the five infectious agents under study (Table 1). Overall, the largest population attributable fractions were found for *H. pylori* and HPV: 2.09% (58 000 cases in 2022) and 1.99% (55 000 cases of whom 40 000 cases were in women), respectively. They were followed by HCV (0.72%; 19 400 cases) and HBV (0.29%; 8100 cases) in both sexes [25]. Figure 2 shows the number of attributable cases by age group, sex and infectious agent. In women, HPV was the most important agent accounting for most cancers caused by infection below the age of 65. In men, *H. pylori* was the most important agent followed by HPV, and HCV while HBV was the rarest. The *H. pylori* population attributable fraction steeply increases with age, becoming the most important cancer‐causing infection in each sex at ages ≥ 75 years.

**Table 1:** Number of cancers attributable to *H. pylori*, HPV, HCV, and HBV in the EU in 2022 and the corresponding population attributable fractions (PAFs) of all cancers attributable to each agent.

Agent	Female		Male		Both sexes	
	*N*	PAF%	*N*	PAF%	*N*	PAF%
**HPV**	40 000	3.14	15 000	0.99	55 000	1.99
***H. pylori***	25 000	1.93	33 000	2.23	58 000	2.09
**HBV**	2100	0.16	6000	0.41	8100	0.29
**HCV**	5400	0.42	14 000	0.99	19 400	0.72
**All**	72 500	5.65	68 000	4.62	140 500	5.09

The following cancer types were considered for each infection: For HPV (Human papillomavirus)—cervical carcinoma, oropharyngeal carcinoma, oral cavity cancer, laryngeal cancer, anal squamous cell carcinoma, penile carcinoma, vaginal carcinoma and vulvar carcinoma. For *H. pylori*—noncardia gastric cancer, and non‐Hodgkin lymphoma of gastric location. For HBV (Hepatitis B virus) —hepatocellular carcinoma. For HCV (Hepatitis C virus) —hepatocellular carcinoma and other non‐Hodgkin lymphomas. The corresponding Population Attributable Fraction (PAF) has been published before [10]. *H. pylori*, *Helicobacter pylori*; HBV, hepatitis B virus; HCV, hepatitis C virus; HIV, human immunodeficiency virus; HPV, human papillomavirus; PAF, population attributable fraction.

**Fig. 3:**
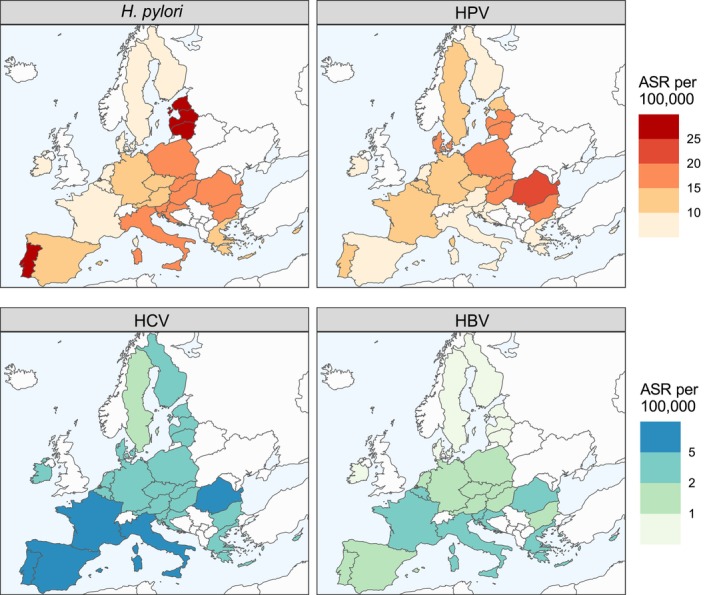
Age‐standardized incidence rates (ASRs) for cancers attributable to *Helicobacter pylori* (*H. pylori*), human papillomavirus (HPV), hepatitis C virus (HCV), and hepatitis B virus (HBV) among men and women combined. ASRs are calculated using the 2022 population in the European Union. Attributable fractions [21] were applied to cancer incidence data from GLOBOCAN 2022 [23] to estimate the ASR attributable to HPV, HBV, HCV, and *H. pylori* infections by country, age, and sex in 2022 [25]. To enable comparison across countries, incidence rates were age‐standardized to the EU population [24]. Although, cancer age‐standardized rates (ASRs) are commonly calculated using the Segi‐Doll world standard population, the standard reflects a much younger age structure than the 2022 EU population. Using it would therefore underestimate the contribution of older age groups, where most cases occur.

Figure 3 shows the heterogeneity in ASRs of cancers due to infections among men and women combined in the EU. For *H. pylori*, the ASR ranged between 6 and 28 per 100 000 individuals with the highest ASRs observed in the Baltic countries and Portugal. HPV‐related cancers show an upward west–east gradient, with the highest ASRs in Central European countries on the eastern border of the EU (ASR range: 5.5–24 per 100 000). Both HBV and HCV showed an upward north–south gradient (ASR range for HBV: 0.4–4 and ASR range for HCV: 2–7 per 100 000), with the highest ASRs in Southern European countries.

## 2 Recommendation for individuals

The ECAC5 experts Working Group (WG) on Infections and related interventions reviewed the most recent scientific literature and updated the ECAC4 recommendation on infections and cancer, following the IARC methodology [4]. As a principle, to modify, adapt or introduce a new recommendation in the ECAC, the current scientific body of evidence should be classified as ‘sufficient’ to demonstrate that adopting the recommendation would lead to reducing an individual's risk of developing or dying from cancer. When including interventions in the recommendation, these should be proven effective to avert or remove the exposure (i.e. infections causing cancer) and/or to avert (progression into) precancerous lesions or cancers. In addition, contextual factors such as equity, suitability, actionability and acceptability of the proposed recommendation in the context of the EU have been addressed by the WG.

### 2.1 Scientific justification for inclusion & update of the recommendation in ECAC5

Infection‐related cancers considered in ECAC5 are all Group 1 carcinogenic to human and can be prevented by (i) preventing individuals from becoming infected; or (ii) treating the infection to slow down or prevent the progression of the disease (e.g. for HBV and HIV) or, ideally, cure the infection (e.g. HCV and *H. pylori*).

The WG evaluated and discussed various sources of published information and recommendations (using EU sources as a priority) on HPV, HBV, HCV, HIV and *H. pylori*. According to the ECAC5 methodology, to introduce a new recommendation when authoritative sources of evidence are not sufficiently recent or not yet available, *ad hoc* systematic literature reviews may be performed by the ECAC5 Literature Group [4]. The WG also relied on systematic reviews previously executed for the 1st edition of the Latin American and the Caribbean Code (LAC) against cancer [26] and three newly requested systematic reviews.

### 2.2 Vaccination

Vaccination is the most efficient way of preventing individuals from becoming infected. Vast evidence shows that vaccination is, among different medical interventions, one of the most feasible, cost‐effective and least prone to social stigma [27, 28].

HBV infection can be prevented through an extremely efficacious and safe vaccine that has been available since the early 1980s. WHO recommends all infants to receive the first dose of hepatitis B vaccine as soon as possible after birth, preferably within the first 24 h [29].

HPV can be prevented by extremely efficacious and safe vaccines (virus‐like particle‐based) that were first licensed in the EU in 2006. Extensive clinical trials and observational studies demonstrated the high efficacy and effectiveness of vaccination in preventing not only HPV infection and precancer [30] but also ICC at the population level [31]. Although several HPV vaccines are now available on the global market, only three vaccines are currently licensed in the EU, that is a bivalent vaccine (2007), a quadrivalent vaccine (2006) and a 9‐valent vaccine (2015), all targeting at least HPV‐16 and HPV‐18. The vast majority of EU countries are currently using the 9‐valent vaccine resulting in one firm having a dominant position in the market. HPV vaccines do not cure already existing HPV infections or HPV‐related disease and therefore WHO recommends administering HPV vaccines before exposure to HPV [27]. Several studies from northern European countries have shown that the effectiveness of the HPV vaccine is highest when administered before the age of 13, with a decline in effectiveness in each successive age cohort starting from age 14 years [32, 33, 34, 35].

Based on previous experience, optimal levels of coverage of HPV vaccination among girls are difficult to achieve in many EU countries (see section ‘Testing and treatment of HBV, HCV, HIV and *H. pylori*’). Vaccinating both genders is in that case the best way to generate strong herd protection against HPV infection in addition to providing direct protection against HPV‐related cancer in men [36, 37]. HPV vaccination in boys has shown similar high efficacy against HPV infection as in girls [38, 39], as also assessed in a systematic review conducted to justify the HPV vaccination recommendation in the 1st edition of the LAC Code [26]. Indeed, gender‐neutral vaccination has been endorsed by all EU countries by 2025 [27]. The WG therefore decided that it is important to include boys in the recommendation on HPV vaccination in ECAC5 in order to emphasize the importance of gender‐neutral vaccination, that is:

*Vaccinate girls and boys against hepatitis B virus (HBV) and human papillomavirus (HPV) at the age recommended in your country.* (*Figure* *1* *)*

### 2.3 Testing and treatment

No large and organized population‐based programmes of testing and treatment of major cancer‐causing infections currently exist in the EU, with the exception of HPV for which there is long‐standing experience in cervical cancer screening [40]. We are using the terminology ‘testing and treatment’ to distinguish our recommendation (which target individuals) from the long‐established population‐based ‘screening’ (i.e. those for cancer of the cervix, breast and colon‐rectum). Different initiatives have been ongoing in the last decades at national and regional level to offer testing and treatment for HBV, HCV, HIV and *H. pylori*, for which strategies target individuals based on symptoms, individual risk or healthcare encounters rather than involving whole‐population outreach strategies.

Since the publication of ECAC4 [2], there have been substantial improvements in the efficacy, safety and accessibility of diagnostic tests and treatments for these infections, especially HCV.

Direct‐acting antivirals (DAAs) against HCV represent a major progress in the field: DAA can achieve a sustained virologic response (SVR) in over 95% of cases, independently of cirrhosis status [15]. Along with a higher cure rate [41], DAAs have the advantage of oral administration and much shorter treatment duration (8–12 weeks) and fewer side effects compared to the previous interferon‐based regimens. Scale‐up of DAA therapy for HCV has led to significant reductions in liver‐related complications, including decompensated cirrhosis and HCC in different world regions [42, 43, 44, 45, 46]. With increasing access to HCV treatment, simplified service delivery models are necessary. A comprehensive systematic review found that task‐shifting to nonspecialist primary care physicians or nurses, achieved HCV cure rates comparable to those of specialist‐delivered care across both general and key populations [47].

HBV treatment cannot eradicate HBV but can suppress viral replication in 70% to 80% of recipients [48, 49]. Tenofovir and entecavir have been shown to be equally effective in reducing the risk of HCC by about 40% [50]. The primary goals of HBV therapy are to reduce morbidity (cirrhosis, hepatic decompensation, liver failure and HCC) and improve survival [49, 51]. A systematic review conducted to justify the HBV testing and treatment recommendation in the 1st edition of the LAC Code showed that in chronic HBV infection, entecavir or tenofovir reduced liver cancer (hazard ratio [HR] 0.44; 95%CI 0.31–0.61) and liver‐related mortality (HR 0.25; 95% CI 0.14 to 0.44). The management of HBV should be especially considered in the presence of comorbidities, notably hepatitis D virus co‐infection, metabolic syndrome, metabolic‐associated steatotic liver disease and type 2 diabetes [52]. The most up‐to‐date clinical practice guidelines on screening, diagnosis and the management of hepatitis B virus infection have recently been published by European Association for the Study of the Liver [51].

The long‐term consequences of HIV infection can almost entirely be prevented by early diagnosis and timely initiation of treatment [53]. PLWH who are diagnosed early and start antiretroviral treatment soon after infection have life expectancies similar to those without HIV [18]. A systematic review conducted to justify the HIV testing and treatment recommendation in the 1st edition of the LAC Code [26] concluded that treatment significantly reduces the risk of Kaposi's sarcoma (KS) and non‐Hodgkin lymphoma (NHL), and also diminishes by approximately 50% the risk of cervical and anal cancer [54, 55].

The efficacy and effectiveness of *H. pylori* testing and treatment approaches in the prevention of gastric cancer, regardless of the presence of symptoms, have been supported by observational studies and randomized clinical trials (RCTs), mainly from Asian countries with historically very high cancer burden [56, 57, 58, 59]. A recently updated meta‐analysis by Ford et al. showed that *H. pylori* eradication among healthy carriers substantially reduced the subsequent incidence of gastric cancer by 36% (RR = 0.64; 95%CI: 0.48–0.84) in 11 placebo‐controlled trials and 44% (RR = 0.56; 95%CI: 0.43–0.73) in 13 observational studies [60]. Of special interest, the meta‐analysis also showed a strong advantage (three trials, RR = 0.52; 95% CI, 0.38–0.71) of *H. pylori* eradication also in infected individuals with gastric dysplasia or early, typically endoscopically detected, gastric cancer. These findings challenge the ‘point of non return theory’ for *H. pylori* testing and treatment possibly expanding the age range in which testing and treatment can be beneficial. However, only two of the studies in the meta‐analysis were conducted outside East Asia, that is one in Colombia (a high‐risk country for *H. pylori*) and one in the United States where gastric cancer incidence is lower than in East Asia [60]. In the EU, a nationwide record‐linkage study from Sweden showed that gastric cancer incidence was significantly reduced in individuals who had undergone *H. pylori* eradication and this effect increased over time since eradication [61]. Of note, strategies of *H. pylori* testing and treatment have been studied for decades and have been reported to be cost‐effective in reducing gastric cancer incidence in high gastric cancer risk areas [62]. However, the real‐world impact of the intervention in the EU is less clear as also mentioned in a recent IARC Working Group Report on the topic [63].

#### 2.3.1 What is the efficacy and safety of treatment of persistent HCV infection to prevent progression to liver disease?

Several clinical trials and observational studies of DAA for persistent HCV infection were published after ECAC4. An *ad hoc* systematic review was therefore requested to update estimates of efficacy and safety of DAAs against HCV [64] (see Annex S2 for overview of PICO questions). The review included four controlled clinical trials and eight observational studies with variable fractions of patients with cirrhosis. Studies comparing DAAs *versus* no treatment showed a reduction of HCC incidence (aHR 0.58; 95%CI: 0.35–0.96 [65] and 0.66; 95%CI: 0.46–0.93 [66]) and liver‐related mortality (aHR 0.39; 95%CI: 0.21–0.71 [65] 0.43; 95%CI: 0.33–0.57 [67]). Studies that compared DAAs to IFN‐based treatment +/− ribavirin showed noninferiority in HCC incidence (aHR varying between 0.53 and 1.47) [65, 68, 69, 70]. Ecological studies showed a decrease in the number of transplantations and patients on waiting lists for the procedure between the pre‐DAA and post‐DAA period.

#### 2.3.2 What is the effectiveness of DAA treatment on persistent HCV infection managed in nonspecialist centres to prevent progression of liver disease?

HCV treatment is relatively new, and it has been mostly offered in specialized‐care centres. An *ad hoc* systematic review was requested to assess the effectiveness of DAA treatment in managing testing and treatment of HCV infection also in nonspecialist centres [71] (see Annex S2, for overview of PICO questions). The review included 12 reports representing six cohorts and six RCTs. Their findings showed that HCV infection managed in nonspecialist centres can increase treatment uptake (pooled RR 1.34; 95%CI 1.10 to 1.64, high certainty) and SVR (pooled RR 1.28; 95%CI 1.02 to 1.62, high certainty) and not diminish treatment completion when compared to specialist centres [72, 73, 74, 75]. The certainty of evidence for improvements in treatment uptake and SVR was high for RCTs, and low for observational studies, possibly due to biases and confounders in the latter ones. Further research to understand the best multidisciplinary protocols to enable testing and treatment in non‐specialist centres should be gathered during testing and treatment programmes in the EU.

#### 2.3.3 What is the effectiveness of screen‐and‐treat for *H. pylori* to prevent progression to gastric disease?

A systematic review was requested on the effectiveness (rather than efficacy, see section ‘Scientific *justification for inclusion & update of the recommendation in ECAC5’* for details on efficacy) of *H. pylori* test‐and‐treat programmes on multiple outcomes in the general population [76] (see Annex S2 for an overview of PICO questions). This review identified two studies conducted in the general population: one from Denmark, based on an *H. pylori* eradication RCT in a community setting, and one from Taiwan, involving community‐based gastric cancer screening integrated with a national colorectal cancer screening programme. *H. pylori* eradication varied between studies: 27% (intention‐to‐treat; RR: 0.73 [95%CI: 0.62–0.87]) [77] and 92% (95%CI: 91.1–92.7) [78]. The effect on gastric cancer incidence, a long‐term outcome, was assessed in a study from Taiwan with a follow‐up of 3 years, which showed a nonsignificant reduction (aRR: 0.91 [95% CI: 0.44–1.89]) [78]. The Danish study with a longer 13‐year follow‐up, although primarily designed to evaluate peptic ulcer disease (PUD), showed no effect on gastric cancer incidence (RR: 1.18 [95% CI: 0.56–2.00]) [77]. A recently updated meta‐analysis by Ford et al., published after the systematic review prepared for ECAC5, has provided new evidence on the time‐window in which testing and treatment can be valid. In fact, *H. pylori* eradication therapy was shown to reduce the incidence of gastric cancer not only in healthy infected individuals but also in patients with early gastric neoplasia [60].

We recommend that additional implementation studies are executed to better understand *H. pylori* testing and treatment interventions in real‐world settings, particularly to identify the most effective modality for implementation in different EU countries. Priorities are those regions where the incidence of gastric cancer is high in the general population or regions in which important high‐incidence minorities exist. In addition, it is important to monitor antibiotic resistance of *H. pylori* in communities in which large‐scale screening programmes are implemented.

Based on available evidence, the working group decided to add a recommendation on testing and treatment of HBV, HCV, HIV and *H. pylori* to the ECAC5, a recommendation that was not present in ECAC4:

*Take part in testing and treatment for hepatitis B and C viruses, human immunodeficiency virus, and* Helicobacter pylori*, as recommended in your country.* *(Figure* *1* *)*

More information on the state of implementation of testing and treatment of HBV, HCV, HIV and *H. pylori* infections in the EU is provided in section ‘Testing and treatment of HBV, HCV, HIV and *H. pylori*’.

### 2.4 Presentation of the recommendation

In summary, the updated ECAC5 recommendation on infections and cancer for individuals of the general public, regardless of the presence of symptoms, is:

*Vaccinate girls and boys against hepatitis B virus (HBV) and human papillomavirus (HPV) at the age recommended in your country.*

*Take part in testing and treatment for hepatitis B and C viruses, human immunodeficiency virus, and* Helicobacter pylori*, as recommended in your country.* *(Figure* *1* *)*

### 2.5 Vaccination of HBV and HPV

#### 2.5.1 Equity

For equity in health care, we intend a situation in which everyone has fair access to essential health interventions according to their needs, independent of their socio‐cultural status. HPV and HBV vaccination uptake varies not only between EU countries but also within countries. National immunization programmes are known to be among the most equitable health intervention if free‐of charge or very cheap. Wide access to HPV and HBV vaccines with gender‐neutral HPV vaccination are anticipated to reduce existing disparities. In certain national programmes of HPV vaccination, there is scope for improving the participation in the general population and/or in marginalized populations or individuals with a migration background [79].

#### 2.5.2 Feasibility

By feasibility in health care, we mean the possibility that something useful can be done, achieved or is reasonable. The resource requirements for individuals vary depending on healthcare systems and vaccination programmes. Generally, EU countries offer HPV and HBV vaccinations free of charge or at a subsidized rate [80].

#### 2.5.3 Acceptability

For acceptability in health care, we refer to the extent to which people and caregivers consider an intervention appropriate based on their anticipated or experienced thoughts and emotions. For decades, large‐scale vaccination programmes have been carried out as an effective way of preventing infectious diseases demonstrating the acceptability of the proposed recommendation. All EU countries have included HBV and HPV vaccination in their national vaccination programmes demonstrating the actionability of the intervention. However, cultural acceptability remains variable, as trust in vaccination is context‐dependent and influenced by political priorities and public perception [80].

Below we describe how these aspects of equity, feasibility and acceptability intertwine with each other in the European context and what actions are needed to overcome existing barriers for implementation.

#### 2.5.4 Current situation in the EU

All EU countries, except two countries with very‐low HBV prevalence (Denmark and Finland), recommend universal vaccination against HBV and all EU countries have implemented universal antenatal screening for the presence of markers of active HBV infection [29]. In the EU/European Economic Area (EEA), that is Iceland, Liechtenstein and Norway in addition to the EU, 10 (39%) of the 25 countries with data are meeting the WHO 2025 target of 95% coverage of HBV vaccination and 14 countries (54%) are within 10% of this set target [29]. Twenty‐six countries reported to also have national HBV vaccination policies focussing on healthcare workers with an HBV vaccination coverage ranging between 20% and 100% [29]. Among the four countries with data on HBV vaccination coverage among people who inject drugs (PWID), coverage ranged from 18% to 54%.

**Fig. 4:**
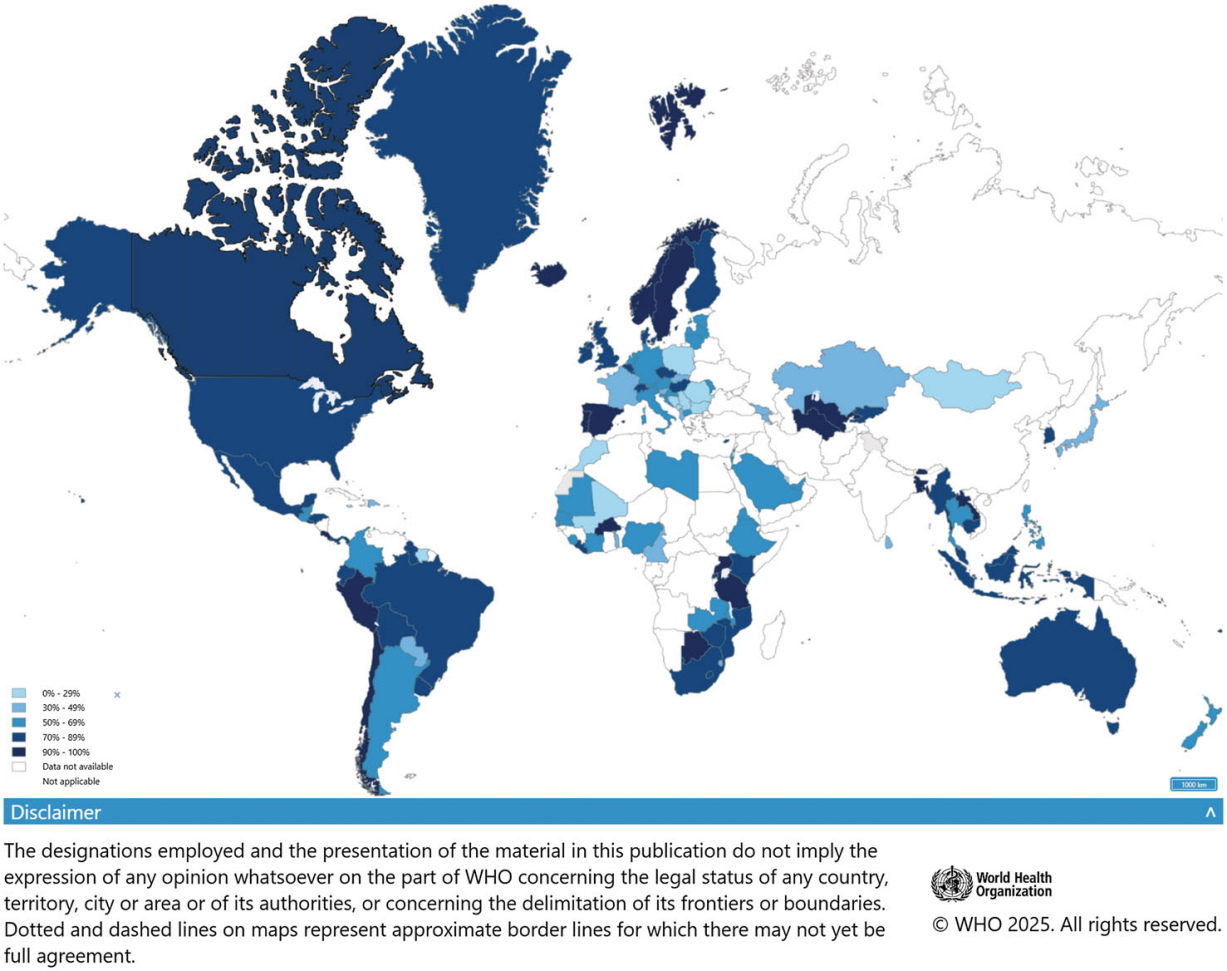
Coverage for first dose human papillomavirus (HPV) vaccination among females by the age of 15 years in 2024 worldwide. Figure obtained from https://www.who.int/teams/immunization‐vaccines‐and‐biologicals/immunization‐analysis‐and‐insights/global‐monitoring/immunization‐coverage.

By 2024, all 27 EU countries introduced vaccination against HPV, but delivery strategy and coverage vary substantially. In girls, HPV vaccination coverage is above 90% in Denmark, Sweden and Portugal, < 50% in five EU countries and not reported in two other countries [81]. Although coverage varies widely across Europe, the intervention has been introduced in most European countries, unlike in many other world regions where it remains largely absent (Fig. 4). Fourteen of the EU countries use a single‐age cohort approach, 13 countries a multi‐age cohort approach, while Germany adopted the widest age range (9–14 years) [81]. HPV vaccination was initially offered only to girls, but by 2024, in all EU countries, the programmes have been extended to include boys, on account of its effectiveness and safety. A few countries (UK, Ireland, Spain and Estonia) have introduced off‐label use of a single‐dose schedule in adolescents, while others continue with two‐dose schedules. HPV vaccination is recommended for children aged 9–14 years, ideally before exposure to the virus, to maximize efficacy. Catch‐up vaccination in age groups beyond the recommended age, particularly in females under 19 years, is cost‐effective and can prevent a substantial number of HPV‐related cancers. Screening will remain necessary for unvaccinated cohorts and for those vaccinated beyond the primary target age (9–14 year) [82] but, over time, guidelines will need to be adapted to suit the lower incidence of HPV infection and its related lesions in vaccinated cohorts.

#### 2.5.5 Necessary actions

National immunization programmes against HBV and HPV are well‐established in the EU but additional efforts are needed to monitor and improve participation in HPV vaccination especially in Eastern EU countries. A prompt identification of any unfavourable trend, possibly due to fake news or organizational challenges, is crucial. While HBV vaccines are cheap, the prices of HPV vaccines are rather high and vary widely across the EU. Although individual cost is usually not a barrier for age cohorts offered vaccines within the national programmes, access to free catch‐up vaccination for older age groups differs between countries and the catch‐up programmes are sometimes time‐limited. Vaccine pricing plays a key role in policy decisions. Evidence shows that centralized, tender‐based procurement can significantly reduce HPV vaccine prices [83]. Expanding such strategies at the EU level could support broader and more equitable access across EU countries.

### 2.6 Testing and treatment of HBV, HCV, HIV and *H. pylori*

#### 2.6.1 Equity

Screening for HBV and HIV in pregnant women is done in all EU countries although with differences in coverage and the proportion of infected women diagnosed. For the general population, there are examples of the offer of testing and treatment for HBV, HCV and HIV in many EU countries. These interventions have primarily been risk‐based, and follow‐up data are limited. Expanding the offer of testing and treatment of cancer‐causing infections in healthcare settings can improve coverage and increase access also for underserved and vulnerable populations (e.g. migrants and people at high risk of acquiring cancer‐related infections), thereby enhancing equity [84]. By recommending testing and treatment, awareness of the issue can increase among individuals and healthcare workers, ultimately contributing to a reduction in the circulation of the infections and of existing inequalities.

#### 2.6.2 Feasibility

The feasibility of testing and treatment for HBV, HCV, HIV and *H. pylori* in EU countries is expected to be good provided the population is well‐informed and the access to the intervention is facilitated. Indeed, high uptake of HIV and HBV testing among pregnant women and blood donors has been demonstrated. Testing and treatment has also been offered to the general population in selected pioneering interventions in the EU [15, 85, 86]. Tests to diagnose HBV, HCV, HIV and *H. pylori* are simple and (mostly) noninvasive. Testing and treatment of HBV, HCV and HIV are relatively inexpensive, and the interventions are likely to be cost‐effective in several EU countries. The cost of new HCV treatment of DAAs may still pose a barrier to HCV treatment in some EU countries, but it can be reduced by, for example, large‐scale purchases. *H. pylori* assays and treatments are widely available at low cost, offering potential healthcare savings.

#### 2.6.3 Acceptability

The acceptability of testing and treatment activities is foreseen to be fairly high as diagnostic assays are not invasive and treatments for HBV, HCV, HIV and *H. pylori* are subsidized or available and affordable in all EU countries. There is still stigma associated with HIV on account of its most common transmission routes, but indicator condition‐based testing can help to result in discreet access to high‐risk individuals. Treatment for *H. pylori* is recommended in many EU countries and concerns over rising antibiotic resistance should be tackled via surveillance studies but should not prevent scale‐up of the intervention in the general population. There is no social stigma around *H. pylori* as it is common and acquired in childhood. The acceptability of testing and treatment of *H. pylori* can be good as shown by the experience of the European Registry for *H. pylori* management [87, 88].

#### 2.6.4 Current situation in the EU

Below, we describe the current European context and the actions that can tackle existing barriers for implementation of testing and treatment of cancer‐causing infections.

For HBV, 15 EU/EEA countries with available data on antenatal screening reported a coverage varying between 57% and 100% (median: 97%). Twelve (80%) countries met the WHO 2025 interim target of 90% coverage of antenatal screening [29]. According to a 2016 ECDC report, 92% (24/26) of EU/EEA countries had implemented HIV antenatal screening [89]. Among the 20 EU countries that provided data on blood donation in 2019, 19 met the WHO 2025 target of screening 100% of blood units for blood‐borne infections.

Apart from antenatal screening for HBV and HIV, no organized national programmes of testing and treating of HBV, HCV, HIV or *H. pylori* are in place in EU countries. A monitoring system for HCV and HBV was developed by the ECDC Prevention and Control [90]. For HCV, three of four countries with available data met the WHO 2025 target of 60% of the estimated number of prevalent cases being diagnosed. Yet, also among these countries, most HCV‐positive persons had not received treatment. For HBV, none of the four countries with data met the WHO 2025 interim target of 60% diagnosed. In the three countries with follow‐up data, the range of people diagnosed with chronic HBV infection who were linked to care was 16–64%.

Data from the WHO European Region (that includes 53 countries including EU/EEA, the Russian Federation and central Asian countries) show that an estimated 72% (64–80%) of PLWH know their HIV status. Of these, 63% (55–70%) are linked to care and on treatment, 60% (53–67%) on treatment achieved viral suppression (HIV RNA <200 copies·mL^−1^) [91]. This does fall short of UNAIDS targets of 95–95–95%. In addition, many PLWH in the EU are still diagnosed at a late stage of HIV infection [92]. Increased testing is crucial for earlier diagnosis, more prompt initiation of treatment, and consequently, better prognosis.

No organized programmes for the detection of *H. pylori* currently exist in EU countries although testing for *H. pylori* among patients with dyspeptic symptoms is a common practice. Despite some downward trends in the last decades, from 2010 to 2022, the overall prevalence of *H. pylori* in adults in the European region was still elevated: 46.7% (95%CI: 40.8–52.7%) (based on 47 studies) [93]. A survey of 34 000 patients was conducted in the framework of the European Registry on the Management of *H. pylori* Infection to assess the use of different *H. pylori* testing methods [88]. In 2013–2021, for the purpose of initial diagnosis, invasive tests (with upper gastrointestinal endoscopy) were performed in 71% of the individuals in the registry, noninvasive tests in 41%, and both in 12%. The detection assays most frequently used were histology (43%), a rapid urease test (38%) and a urea breath test (27%). Invasive tests were performed in 77% of individuals aged ≥ 50 years and in 65% in those aged < 50 years. These real‐world data show that the rapid urease test was the most common test used to confirm the eradication of *H. pylori* infection, while in general noninvasive tests (i.e. urea breath test [UBT] or fecal antigen tests) are recommended. Clinicians should refer to the Maastricht VI/Florence consensus report on the most up‐to‐date clinical guidelines for the EU [94] and the last Working Group Report from IARC [63]. The volume includes comprehensive information on the detection methods, treatment options and other considerations about antibiotic stewardship, target groups, including an up‐to‐date list of international guidelines on a number of detailed aspects of screening *H. pylori* and treatment for gastric cancer prevention, for example modalities of diagnosis, treatment and follow‐up [63].

#### 2.6.5 Necessary actions

ECDC provides guidance for testing strategies that can be implemented within existing healthcare settings in EU countries to enhance coverage and diagnosis: (a) population prevalence‐based testing, (b) birth‐cohort testing and (c) indicator condition‐guided HIV testing [95]. The WHO emphasizes that key decisions in any testing and treatment programme to be made are: When (frequency and timing); Where (location of mobilization); Who is providing service (health workers, peers, or self‐testing); and What (what to include in the package of offered services) [86].

Integrated programmes of concurrent testing and treatment for more than one cancer‐associated infection may further increase the feasibility and cost‐effectiveness of the intervention. As an example, in the United Kingdom, a country with low HCV and HBV prevalence (0.14% and 0.6% in 2022, respectively), an opt‐out testing programme for HBV, HCV and HIV among all adults presenting to emergency departments was implemented. This initiative led to increased diagnosis rates, reduced transmission, improved health outcomes and the normalization of blood‐borne virus testing in emergency settings [86]. In the United States in 2012, the CDC recommended one‐time HCV screening to all baby boomers (individuals born between 1945 and 1965) [96]. Preliminary data indicate that only 4.0–12.9% of baby boomers had previously been offered an HCV test by a healthcare professional [97]. A record‐linkage study from a large academic health system found that among patients offered an HCV antibody test, compliance with treatment was 73%, with minimal variation by age, sex or ethnicity [97]. In 2020, the CDC expanded this recommendation to all adults and for all pregnant women during each pregnancy, except in settings where HCV prevalence is below 0.1% [96]. In 2023, a similar strategy was introduced for HBV by the CDC [98]. Of note, experiences from high‐endemic countries, for example Egypt, have shown that mass screening and immediate treatment can significantly reduce disease burden [86, 99].

In respect to the current performance of testing and treatment of *H. pylori* in the EU, the findings of the above‐mentioned *H. pylori* Infection Registry showed that EU gastroenterologists perform upper gastrointestinal endoscopy for the initial diagnosis of *H. pylori* in approximately half of the patients below age 50 years, in whom it is not recommended [88]. Conversely, an upper gastrointestinal endoscopy tends to be often omitted in potentially high‐risk patients older than 50 years. Several studies on gastric cancer prevention through *H. pylori* screen‐and‐treat strategies are under way in Europe [63]. In particular:

- The GISTAR study: a multicentre randomized trial in Latvia that is focussing on *H. pylori* eradication and pepsinogen testing as a method to reduce gastric cancer mortality in middle‐aged people [100].
- Accelerating Gastric Cancer Reduction in Europe through *H. pylori* Eradication (EUROHELICAN), supported by the EU4Health programme, to assess the feasibility, acceptability and effectiveness of implementing a population‐based *H. pylori* screen‐and‐treat programme in young adults (aged 30–34 years) in Slovenia [101].
- Towards Gastric Cancer Screening Implementation in the European Union (TOGAS) study, also supported by the EU4Health programme, evaluates three approaches to gastric cancer screening: (I) *H. pylori* screen‐and‐treat in young adults (30–34 years); (ii) upper endoscopy in individuals undergoing colorectal cancer screening or surveillance colonoscopy; and (iii) long‐term impact of *H. pylori* eradication in the GISTAR cohort (combining *H. pylori* and pepsinogen testing) [102].
- European Implementation Study on simultaneous screening for gastric and colorectal cancers within EUCanScreen to address the potential of screening and treatment for *H. pylori* at the time of initiating colorectal cancer screening with a fecal immunochemical test (FIT).

These ongoing studies should provide guidance on further implementation of *H. pylori* screening/testing and treatment approaches in the EU, including the number needed to treat in different EU countries and potential harms like increases in antibiotic resistance. In addition, the upcoming European Commission Initiative on Gastric Cancer will develop guidelines on *H. pylori* screen‐and‐treat for gastric cancer prevention in Europe.

Population‐based *H. pylori* testing and treatment could significantly reduce gastric cancer incidence, particularly in high‐risk European regions and subpopulations (Fig. 3). Multidisciplinary protocols and resources for primary care providers are crucial to introduce feasible and affordable testing and treatment approaches for *H. pylori*. In particular, the tasks that should be handled by primary care physicians and by gastroenterologists in specialized centres should be established according to the target population and national context. In 2025, IARC's working group published a report with further guidance on the implementation of population‐based *H. pylori* screen‐and‐treat strategies for gastric cancer prevention [63].

### 2.7 Cobenefits for diseases other than cancer with similar risk factors and opportunities for health promotion

Diseases other than cancer that could be prevented by vaccination against HPV include genital warts (mainly from HPV‐6 and ‐11) and recurrent respiratory papillomatosis. HBV vaccination and testing and treatment of HBV and HCV can prevent chronic liver disease, cirrhosis and progression of metabolic dysfunction‐associated fatty liver disease (both viruses), or type 2 diabetes (mainly HCV). PLWH have an increased risk of developing cardiovascular and neurological disorders that is reduced but not eliminated by ART. *H. pylori* is associated with peptic ulcer disease, *H. pylori* gastritis‐associated dyspepsia, iron‐deficiency anaemia, vitamin B12 deficiency, mucosa‐associated lymphoid tissue gastric lymphoma and idiopathic thrombocytopenic purpura. In addition, many other risk factors (e.g. alcohol consumption, tobacco smoking, excess body weight or other noncommunicable diseases such as diabetes) work in conjunction with these infections exacerbating the risk of infection‐related cancers. For example, a weakened immune system from HIV, cancer treatment or organ transplantation can predispose individuals to HPV‐related cancers. Preventing these infections may therefore mitigate the joint effects of multiple risk factors on disease development.

## 3 Recommendation for policymakers

Presentation of the recommendations for policymakers and key stakeholders (e.g. health professionals). HPV, HBV, HCV, HIV and *H. pylori* are responsible for ~ 5% of cancer cases in the EU and for higher proportions in some countries in Southern and Eastern Europe. Importantly, many infection‐related cancers can be avoided using available interventions. Indeed, our recommendations for reducing cancer risk through preventive health interventions are grounded in key EU and international policies that emphasize vaccination [27, 29, 103, 104, 105, 106] and early intervention for infection‐related cancers [63, 84, 94, 105, 106, 107, 108], including overarching policy documents [109, 110]. These documents guide our recommendations to strengthen vaccination programmes and introduce sustainable testing and treatment strategies for five major cancer‐related infections. Therefore, we propose making preventive health interventions the default option, facilitating healthier choices for individuals across EU countries. See Table 2, for details on recommendations for policymakers and key stakeholders.

**Table 2:** European Code Against Cancer, 5th edition: recommendations for policymakers on cancer‐causing infections and related interventions.

Cancer‐causing infections and related interventions
**Strengthen hepatitis B virus (HBV) vaccination programmes to maximize their effect on reducing the prevalence of HBV infection. This can be achieved, according to the epidemiological burden, by ensuring that:** ○ All children receive their first dose of HBV vaccine as soon as possible after birth. ○ HBV vaccination programmes are resourced to reach the 95% coverage target. ○ Catch‐up vaccinations are offered to people at increased risk of acquiring HBV infection. **Strengthen human papillomavirus (HPV) vaccination programmes to maximize their impact by ensuring that:** ○ The vaccine is given at the youngest age possible to the priority target age group (between 9 and 14 years) as decided at the national level. ○ HPV vaccination programmes are resourced to reach the 90% coverage target for girls and boys. ○ Catch‐up vaccination opportunities are provided to people older than the priority target age but at least until age 18 years, when feasible. ○ Individuals at high risk of HPV infection, including immunocompromised individuals and people who experienced sexual abuse, are considered for vaccination against HPV as a priority. Individuals known to be immunocompromised or infected with human immunodeficiency virus (HIV) should receive at least two HPV vaccine doses and, where possible, three doses. **Strengthen the importance of HBV and HPV vaccination as cancer prevention tools** ○ This includes identifying behavioural determinants of vaccine uptake, addressing obstacles to vaccination and implementing awareness‐raising campaigns to increase confidence in these vaccines among health professionals, teachers, parents and (pre) adolescents. ○ Monitor progress in vaccination programmes against HBV and HPV in a timely manner. **Introduce sustainable initiatives of testing and treating:** ○ Adopt policies facilitating the offer of an affordable, ideally free of charge, test for HBV, hepatitis C virus (HCV), HIV, and *Helicobacter pylori* (*H. pylori*) to adults in low‐threshold settings using a non‐stigmatizing approach. ○ Treat individuals with confirmed HCV, HIV, or *H. pylori* infection as early as possible. For HBV, treatment should be provided to selected individuals, according to the clinical guidelines. ○ Offer pregnant women HBV and HIV testing, and consider offering also HCV testing based on individual risk assessment. ○ For HIV, the offer of testing should prioritize individuals with HIV indicator conditions and people at increased risk of sexual acquisition of HIV or exposure to blood and blood products. ○ Develop and coordinate public health awareness campaigns related to all infections that cause cancer and interventions that avoid their acquisition or progression to disease. ○ Monitor progress in test‐and‐treat strategies in the population, including low‐literacy and vulnerable groups.

© 2026 International Agency for Research on Cancer / WHO. Used with permission. References: • Council recommendation of 21 June 2024 on vaccine preventable cancers. *OJEU*. 2024;**C4259**:1–8. Available from: https://eur-lex.europa.eu/eli/C/2024/4259 [103]. • Council recommendation on strengthening prevention through early detection: a new EU approach on cancer screening replacing Council Recommendation 2003/878/EC. Brussels: European Commission; 2022. Available from: https://eur-lex.europa.eu/legal-content/EN/TXT/?uri=celex:32022H1213(01) [108]. • European Centre for Disease Prevention and Control (ECDC). Prevention of hepatitis B and C in the EU/EEA. Stockholm: ECDC; 2022. Available from: https://www.ecdc.europa.eu/assets/Prevention-Hepatitis-B-and-C/index.html [104]. • Public health guidance on HIV, hepatitis B and C testing in the EU/EEA: an integrated approach. Stockholm: European Centre for Disease Prevention and Control (ECDC); 2018. Available from: https://www.ecdc.europa.eu/sites/default/files/documents/hiv-hep-testing-guidance_0.pdf [84]. • Regional action plans for ending AIDS and the epidemics of viral hepatitis and sexually transmitted infections 2022‐2030. Copenhagen: WHO Regional Office for Europe; 2023. Available from: https://iris.who.int/bitstream/handle/10665/369243/9789289058957-eng.pdf?sequence=7 [105]. • Guidelines for the prevention, diagnosis, care and treatment for people with chronic hepatitis B infection. Geneva: World Health Organization; 2024. Available from: https://iris.who.int/bitstream/handle/10665/376353/9789240090903-eng.pdf?sequence=1 [106]. • Human papillomavirus vaccines: WHO position paper (2022 update). Geneva: World Health Organization; 2022. Available from: https://www.who.int/publications/i/item/who-wer9750-645-672 [27]. • Park JY, editor Population‐based Helicobacter pylori screen‐and‐treat strategies for gastric cancer prevention: guidance on implementation (IARC Working Group Reports No. 12). Lyon, France: International Agency for Research on Cancer; 2025. Available from: https://publications.iarc.who.int/648. Licence: CC BY‐NC‐ND 3.0 IGO. [63].

To prevent the acquisition of HPV and HBV, we recommend gender‐neutral vaccination through national health care programmes in line with the recommendations from the European Commission [103], and the ECDC [29, 104]. Similarly, WHO advocates integrating HPV vaccination into national strategies to eliminate cervical cancer and reduce tumours of the anogenital tract and oropharynx [5]. Catch‐up HPV vaccination up to age 18 years is recommended when feasible, as many adolescents remain unvaccinated at the routine target age and may still benefit from protection before exposure. Individuals at increased risk of HPV‐related disease, including immunocompromised persons and those with a history of sexual abuse, should be prioritized for vaccination, with at least two doses and ideally three doses recommended for those with immunosuppression, including HIV infection, to ensure adequate immunogenicity. By making HPV and HBV vaccination the default preventive measure within healthcare settings, the EU can establish environments that support equitable access, including underserved populations.

Implementing testing and treatment strategies for HBV, HCV, HIV, and *H. pylori* is also necessary to reduce the burden of infection‐related cancers. Section ‘Testing and treatment of HBV, HCV, HIV and *H. pylori*’ includes further details on the current situation and necessary actions for implementation. ECDC's Public Health Guidance recommends integrated testing and treatment of HIV, HBV and HCV [84]. Expanding HBV, HCV and HIV testing and treatment aligns with WHO's elimination goals, supporting early diagnosis and reducing cancer risks (71). The Maastricht VI/Florence Consensus Report provides detailed guidance on standardized approaches for *H. pylori* eradication [94] and the last IARC's Working Group Report provides strategies for population‐based *H. pylori* screen‐and‐treat strategies for gastric cancer prevention [63]. In addition, clear endorsement of population‐based testing and treatment for major cancer‐related infections also comes from the European Commission's 2022 Council Recommendation on Strengthening Prevention Through Early Detection [107, 108, 109].

Integrating programmes of vaccination (HBV and HPV) and testing and treatment of HBV, HCV, HIV and *H. pylori* within existing healthcare systems can also improve the health of underserved populations and reduce healthcare disparities. Essential questions that should be answered according to the local situation include: When (frequency and timing of the intervention); Where (location of mobilization); Who is providing service (health workers, peers, or self‐testing); and What (which package of services has to be offered) [86]. Consistent with the European Commission's Beating Cancer Plan [109], our recommendations aim to prioritize sustainable health systems and preventive care within primary care and public health settings.

## 4 Conclusions

Five infections are an important cause of preventable cancer in the EU. While the burden is much higher in Southern and Eastern European countries than elsewhere in the EU, subpopulations with substantial prevalence of cancer‐associated infections are present in all EU countries. The introduction of routine screening of blood donors for HBV, HCV and HIV and the use of disposable needles since the 1990s have curbed the iatrogenic transmission of these blood‐borne viruses but vaccinations and testing and treatment are increasingly affordable, crucial to further diminish the circulation of and the disease burden caused by these infections.

The success of all these tools is highly dependent on political will and public trust in their efficacy and safety [111]. By combining efforts on HBV, HCV and HIV testing and treatment, countries may tackle these infections in the most cost‐effective way, in line with ECDC's advice [84]. Furthermore, testing and treatment of *H. pylori* could significantly reduce gastric cancer incidence, particularly in high‐prevalence European regions and subpopulations at increased risk because, for example, place of birth [63]. The increase in experience due to the gradual introduction of testing and treatment of cancer‐associated infections expected from the new recommendation in ECAC5 will provide further guidance on the best protocol to be used in general healthcare setting, hopefully evolving in the implementation of population‐based screening programmes. EU‐wide monitoring of progress in vaccination and testing and treatment of cancer‐causing infections is crucial to carry out the current recommendations.

## Conflict of interest

MSL and CJA's institution receives study funding from GSK for an investigator‐initiated study on HPV vaccination, MSL served on an advisory board of Novosanis; all payments were made to his institution. FN previously received consulting fees and honoraria from Gilead Sciences, with the last contact ending in October 2023. He has had no financial or professional relationships with industry since that time. PM has received compensation in the form of advisory board fees or speaker honoraria in sponsored symposia, most recently in 2022, from the following companies: Aboca, Allergosan, Bayer, Biocodex, Biohit, Cinclus, Malesci, and Menarini. PB, SS, SG, ML, PM, MM, FM, FN, MP, MSL, GB, JS, HZ, AF, ES, DR, CE declare no conflict of interest. Where authors are identified as personnel of the International Agency for Research on Cancer/World Health Organization, the authors alone are responsible for the views expressed in this article and they do not necessarily represent the decisions, policies, or views of the International Agency for Research on Cancer/World Health Organization.

## Author contributions

SF and CJA drafted the article and adapted this version after in‐depth discussions with all the co‐authors, other experts and the IARC Secretariat. MP estimated the burden of infection‐associated cancer in the EU. All the authors read and approved the final version of the manuscript.

## Supporting information


**Annex S1.** European Code Against Cancer, 5th edition. © 2026 International Agency for Research on Cancer / WHO. Used with permission.

**Annex S2.** PICO questions for Systematic Reviews requested by Working Group 3 (ECAC5).

## Data Availability

The data that support the findings of this study are available in Table 1, Figs 2, 3, 4, and/or the supplementary material of this article. The 5th edition of the European Code Against Cancer (ECAC5) contains 14 recommendations on cancer prevention. Here, we update the cancer prevention recommendations related to cancer‐causing infections, namely *H. pylori*, HPV, HBV, HCV and HIV, positioned as recommendation number 12. ECAC5 recommends: (a) vaccinate girls and boys against HBV and HPV at the age recommended in your country; and (b) take part in testing and treatment for HBV, HCV, HIV and *H. pylori*, as recommended in your country. Policymakers should expand vaccination and improve access to testing and treatment.
