# Supplementary material for: European Code Against Cancer, 5th edition – cancer‐causing infections and related interventions
Source: Mol Oncol. 2026 Jan 16;20(1):96–116. doi: 10.1002/1878-0261.70172 (PMC12809466; doi:10.1002/1878-0261.70172)
Supplement: Supplementary file 2 — Annex S2. PICO questions for Systematic Reviews requested by Working Group 3 (ECAC5). [file MOL2-20-96-s001.docx]

**Annex II – PICO questions for Systematic Reviews requested by Working Group 3 (ECAC5)**

**PICO for the Systematic Review on the research question: What is the effectiveness of DDA treatment of persistent HCV infection managed in non-specialist centres to prevent progression to liver disease?**

| **Population** |
| --- |
| General population.  Adult patients (18 years and older) with chronic hepatitis C virus infection (diagnosed by hepatitis C virus ribonucleic acid [HCV RNA] or hepatitis C virus core antigen), with no evidence of decompensated cirrhosis and/or liver cancer, and undergoing treatment with direct-acting antiviral agents. |
| **Intervention** |
| Provision of hepatitis C virus treatment in any primary care setting (defined by the World Health Organization as “first contact of accessible, continued, comprehensive and coordinated care”) or in community environments (i.e. settings where participants live), using any direct-acting antiviral therapy. The care provider could be any type of health care professional. |
| **Comparison** |
| Care in any hospital or secondary care environment with the same type of treatment regimes. |
| **Outcomes** |
| *Primary outcome:*   - Treatment uptake - Treatment completion - Sustained virologic response (SVR)   *Secondary outcomes*   - Relapse - Cirrhosis (defined as compensated versus decompensated if possible) - Liver cancer (hepatocellular carcinoma; HCC) - Liver cancer mortality - Liver transplantation (+/- mortality) - Adverse events |

**PICO for the Systematic Review on the research question: What is the efficacy and safety of treatment of persistent HCV infection to prevent progression to liver disease?**

| **Population** |
| --- |
| Adult patients with chronic hepatitis C virus infection (diagnosed by HCV RNA or HCV core antigen) |
| **Intervention** |
| Direct-acting antiviral agents includingNS3/4A protease inhibitors, NS5A  protein inhibitors, NS5B nucleoside (NPIs), and nonnucleoside (NNPIs)  polymerase inhibitors administered for at least 8 weeks and delivered in  the following fixed-dose combinations:  -Sofosbuvir/daclatasvir  -Paritaprevir/ritonavir-ombitasvir &amp; dasabuvir  -Sofosbuvir/velpatasvir  -Ledipasvir/sofosbuvir  -elbasvir/grazoprevir  -glecaprevir/pibrentasvir  -Sofosbuvir/velapatasvir/voxilaprevir  -Simeprevir/sofosbuvir  -Paritrapevir/ritonavir-ombitasvir  -Asunaprevir/daclatasvir/beclabuvir |
| **Comparison** |
| -Usual care (pegylated interferon alfa, ribavirin)  -Placebo or no treatment |
| **Outcomes** |
| Primary outcome:  - Cirrhosis (defined as compensated versus decompensated if possible)  - Liver cancer (hepatocellular carcinoma; HCC)  - Liver cancer mortality  - Liver transplantation (+/- mortality)  Secondary outcomes  - Sustained virologic response (SVR)  - Relapse  - Adverse events |
| **Design** |
| Prospective or retrospective cohorts of at least 100 participants |

Secondary outcomes were reported only when provided by studies that also assessed the primary outcomes. Studies involving mixed populations (e.g., with and without decompensated cirrhosis) were included only if they presented stratified analyses relevant to the target population.

**PICO for the Systematic Review on the research question: What is the effectiveness of screen- and-treat for *H. pylori* to prevent progression to gastric disease?**

| **Population** |
| --- |
| General population |
| **Intervention** |
| Screen (i.e., urea breath test, serologic or stool tests) and treat strategies for *H. pylori* (any treatment scheme). |
| **Comparison** |
| No screening |
| **Outcomes** |
| *Primary outcome:*   - Incidence of gastric cancer (adenocarcinoma or Mucosa-Associated Lymphoid Tissue [MALT]) confirmed by endoscopic biopsy and histology. - Gastric cancer mortality - Overall mortality   *Secondary outcomes*   - Successful eradication of *H. pylori* (confirmed by breath-test and faecal antigen test) - Cure rate of peptic ulcer - Short and long-term adverse events (e.g., diarrhoea, proportion of individuals stopping therapy because of adverse events, microbiome alterations etc.) |
